# Organizational Prevention and Management Strategies for Workplace Aggression Among Child Protection Workers: A Project Protocol for the Oslo Workplace Aggression Survey (OWAS)

**DOI:** 10.3389/fpsyg.2020.01401

**Published:** 2020-06-30

**Authors:** Morten Birkeland Nielsen, Jan Olav Christensen, Jørn Hetland, Live Bakke Finne

**Affiliations:** ^1^National Institute of Occupational Health, Oslo, Norway; ^2^Department of Psychosocial Science, University of Bergen, Bergen, Norway

**Keywords:** violence, aggression, harassment, prevention and management, safety, health

## Abstract

**Background:**

Previous research has established exposure to workplace aggression as a significant risk factor for employee functioning, well-being, and health. However, less is known about effective prevention and management strategies. The main objectives of the current project were to determine the impact of physical and psychological aggression on the well-being, health, and work ability of employees in the child welfare service and to establish whether a strong psychosocial safety climate and an ethical infrastructure are effective with regard to protecting employees against aggression. This project may help identify the specific risks child welfare workers are exposed to, the impact of workplace aggression on their health and well-being, and the most effective strategies to manage the problem. Furthermore, the findings should be central for developing laws and regulations and to any political decision on measures to tackle aggression in the workplace.

**Methods:**

The study will employ two prospective data collections. Firstly, a three-wave longitudinal survey with a 6-month time lag between measurement points will be conducted among all 1,500 employees in the child welfare services in Oslo Municipality, Norway. Data will have a multilevel structure and will be linked to registry data on sickness absence. Secondly, a quantitative daily diary study over a 14-day period will include 150 of the respondents from the main survey study. The survey questionnaires mainly comprise well-established and psychometrically validated indicators of workplace aggression, health and well-being, psychosocial safety climate, ethical infrastructure, and other relevant factors. The Regional Committees for Medical and Health Research Ethics (REC) in Norway (REC South East) have approved this project (project no. 28496).

**Discussion:**

This project will identify the impact of workplace aggression on child protection workers as well as provide information on how organizations can actively manage exposure to workplace aggression. The findings may serve as a starting point for intervention studies as well as the development of policies and guidelines on how to handle workplace aggression.

## Introduction

Child welfare work is concerned with ensuring the welfare and well-being of children by assisting parents in giving their children the best possible upbringing. However, working with children, and especially in cases of maltreated children, is also a risk factor for child welfare workers’ own psychological well-being (Baugerud et al., 2018). In addition to having to deal with cases that are challenging and even traumatic, many employees in the child welfare service are exposed to physical and psychological aggression from both the clients and their relatives (Littlechild, 2005). With previous research showing that a stressful working environment is a main cause of bullying and harassment (Hauge et al., 2007; Van den Brande et al., 2016), employees in the child welfare service are also at increased risk of being exposed to aggression from co-workers.

Workplace aggression involves experiencing behavior that (1) is potentially harmful, (2) the target is motivated to avoid, and (3) occurs while the target is working (Schat and Frone, 2011, p. 24). The two main categories of workplace aggression are *physical violence* and *psychological aggression*. Physical violence involves behavior characterized by a physical act where the typical immediate and primary effect is physical harm. Examples of physical violence are beating, kicking, slapping, stabbing, pushing, biting, and pinching, as well as direct threats of such behaviors. Psychological aggression involves behavior characterized by a verbal or symbolic act where the typical immediate effect is psychological harm (Schat and Frone, 2011). Psychological aggression can often be indirect and abstract and can include verbal abuse, ostracism, slander, humiliation, unreasonable criticism, and bullying. When seen from the perspective of the perpetrator, differentiating between hostile aggression and instrumental aggression is also common (Anderson and Bushman, 2002): Hostile aggression is conceived as being impulsive, thoughtless (i.e., unplanned), driven by anger, having the ultimate motive of harming the target, and occurring as a reaction to some perceived provocation. Instrumental aggression is conceived as a premeditated means of obtaining some goal other than harming the victim and being proactive rather than reactive. In the present project, we will, however, only investigate workplace aggression as seen from the target perspective. The focus will therefore be on the individual consequences of being exposed to aggression at the workplace rather than on identifying the motives or intent behind the perpetrated aggression.

The transactional model of stress and coping (Lazarus and Folkman, 1984) and the shattering of basic assumptions theory (Janoff-Bulman, 1992) are theoretical frameworks that can explain the individual consequences following exposure to workplace aggression. According to the transactional model, the ability to cope with negative life events, such as aggression, is determined by two consecutive appraisal processes. The primary appraisal process consists of the cognitive appraisal of the adversity of the aggressive situation for its potential for harm or loss. If the target perceives the situation as threatening, a secondary appraisal process is initiated, centering on whether one has available options or adequate resources to handle the aggression in order to prevent the threat of harm or loss. If individuals perceive that the exposure is taxing or exceeding the available options and resources, the transactional model proposes that individuals experience strain (i.e., an imbalance between demands and resources). Strain over an extended time period may produce mental distress (e.g., anxiety, depression, and exhaustion), which, again, can develop into somatic complaints and disorders (Watson and Pennebaker, 1989). Janoff-Bulman’s (1992) theory on “shattered basic assumptions” extends the transactional model by providing a mechanism that can explain how workplace aggression can impact the health and well-being of those exposed. According to Janoff-Bulman’s theory, aggression is likely to break down the targets’ assumptions of themselves as valuable and worthy individuals, of other people as benevolent, and of the world as meaningful (Mikkelsen, 2001; Mikkelsen and Einarsen, 2002). Such abrupt changes in the core conceptual beliefs about ourselves and the world are threatening and can inflict worrying, rumination, and psychological crisis, which subsequently lead to mental disorders (Janoff-Bulman, 1992).

As for specific health consequences following workplace aggression, several systematic reviews and meta-analyses show that those exposed are at significantly higher risk of developing mental and somatic health complaints (Lanctot and Guay, 2014; Nielsen et al., 2014; Rudkjoebing et al., 2020), symptoms of posttraumatic stress (Jacobowitz, 2013; Nielsen et al., 2015b), and suicidal ideation (Leach et al., 2017). Targets also report higher turnover and lower affective commitment (LeBlanc and Barling, 2004; Bowling and Beehr, 2006), lower productivity (Bowling and Beehr, 2006), reduced work ability as manifested through higher sickness absence rates (Nielsen et al., 2016), and risk of disability retirement (Nielsen et al., 2017a). A prospective study of female health and social workers in Norway found that employees exposed to threats and violence had a 67% increased risk of long-term medically certified sickness absence (Aagestad et al., 2014). Hence, work-related aggression has significant costs for both individuals, employers, stakeholders, and the society (Lanctot and Guay, 2014; Hassard et al., 2017), and it has been estimated that workplace aggression represents an annual financial burden for society ranging between US $114.64 million and US $35.9 billion (Hassard et al., 2017).

These extensive costs suggest that knowledge regarding how employees can be protected against the impact of workplace aggression is highly important. As previous research has shown that personal resources only have a protective effect in cases of no or only low exposure to aggression, and not in cases of high exposure (Nielsen and Einarsen, 2018), resources provided by the employer seem to be especially vital with regard to counteracting the impact of workplace aggression. One such resource is psychosocial *safety climate*, which refers to the organizational policies, practices, and procedures for the protection of workers’ psychological health and safety (Dollard and Bakker, 2010). In contrast to *safety culture*, which encompasses a set of shared beliefs, values, attitudes, and customs concerning workplace safety, safety climate is the perceived value placed on safety in an organization at a particular point in time (Christian et al., 2009). Hence, safety climate can be considered as a snapshot, and thereby a measureable component, of an organization’s culture. A closely related concept is “ethical infrastructure,” which consists of the formal and informal systems, each including communication, surveillance, and sanctioning components, that are used to counteract workplace aggression (Tenbrunsel et al., 2003; Einarsen et al., 2017). Due to its focus on safeguarding employees, ethical infrastructure constitutes the basis for psychosocial safety climate. It appears that when ethical systems are present, the psychosocial safety climate is likely to be perceived as stronger (Einarsen et al., 2017), and it is therefore important to assess both phenomena in order to understand how organizations can protect employees against the impact of workplace aggression.

Based on the main assumptions of the “psychosocial safety climate model” (PSCM; Dollard et al., 2012), a workplace with a strong psychosocial safety climate and an ethical infrastructure should have available policies and procedures that actively manage psychosocial risk factors. Human resource divisions, health and safety personnel, and managers should have clear methods for the promotion and protection of worker health and well-being. Employees are encouraged to utilize the available tools to improve well-being and to report incidences of aggression (Dollard et al., 2007; Dollard et al., 2011). In order to be effective, all levels of the organization (executive, management, and worker) need to have implemented these policies, procedures, and practices relating to health and well-being. However, leaders and the management seem to play an especially important role. Evidence shows that both top-level management and supervisor-level management serve as role models that can influence employee behavior (Zohar and Luria, 2003, 2005; Walumbwa and Hartnell, 2011), and leaders are also highly important with regard to shaping the organizational culture (Schein, 1990). In the context of workplace aggression, leaders may directly prevent employees from future exposure to aggression, for instance by considering prevention in decisions concerning staffing and the intake of clients (Gadegaard et al., 2018), and they may also contribute to buffering the effect of aggression if employees perceive that cases of aggression are handled effectively. Hence, in the perspective of the PSCM, a strong psychosocial safety climate and an ethical infrastructure function as preeminent protective factors against the occurrence of workplace aggression while also being protective resources against negative outcomes if aggression does occur (Dollard and Bakker, 2010). This means that having a strong psychosocial safety climate and an ethical infrastructure should reduce the occurrence and, in addition, alleviate the consequences of aggression.

### Objective and Research Questions

**FIGURE 1:**
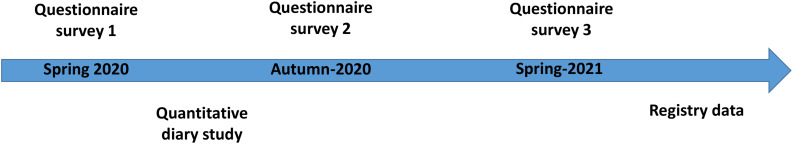
Conceptual model for the research project (direct and indirect associations indicated by *blue lines* and moderation effects indicated by *black lines*).

In this project, we will empirically examine the role of ethical infrastructure and psychosocial safety climate with regard to exposure to multiple forms of workplace aggression among child welfare workers. The forms of aggression that will be investigated can stem from both clients and colleagues and include physical aggression, as displayed through verbal and physical threats and actual physical violence, and psychological aggression, as displayed through verbal abuse, threats to family members, online harassment, and bullying. The main reason for examining multiple forms of workplace aggression from different aggressors (e.g., clients and colleagues) is that both the nature of the aggression and the identity of the aggressor are likely to result in different outcomes. Consequently, specific intervention strategies will be needed for each form of aggression. Figure 1 provides a graphical overview of the associations that will be examined in the project. Specifically, we will investigate how the psychosocial safety climate and ethical infrastructure influence the risk of physical and psychological aggression, as well as the outcomes of these exposures, among child welfare workers. We expect that workers with a strong psychosocial safety climate and with the ethical infrastructure in their workplace are at lower risk of workplace aggression and that these workers have a lower risk of health complaints if experiencing aggression.

In order to understand the health effects of workplace aggression, it is also important to identify the mechanisms that determine how workplace aggression creates health problems. To this date, only a few studies, mainly based on cross-sectional self-report data from small and specific samples, have examined such explanatory variables (Neall and Tuckey, 2014; Nielsen and Einarsen, 2018; Rudkjoebing et al., 2020). In this project, we will therefore examine these mechanisms applying longitudinal data. These kinds of prospective studies have been requested in the literature on workplace aggression (Rudkjoebing et al., 2020). Based on the theory of shattered basic assumptions described above (Janoff-Bulman, 1989; Mikkelsen and Einarsen, 2002; Nielsen and Einarsen, 2018), it is likely that workplace aggression influences the health and well-being of those exposed through factors such as worrying (Rosario-Hernández et al., 2018; Demsky et al., 2019), job dissatisfaction (Devonish, 2013), psychological detachment (Demsky et al., 2014, 2019), and job engagement (Einarsen et al., 2018). The following primary research questions will be addressed:

1. What is the prevalence of physical and psychological aggressions among employees in the child welfare service?
2. To what extent do the psychosocial safety climate and ethical infrastructure influence the occurrence of physical and psychological aggression?
3. How and when do physical and psychological aggressions impact the health and well-being of employees in the child welfare service?

The following general hypotheses will be tested with regard to the third research question:

3a. Worrying, job dissatisfaction, psychological detachment, and job engagement mediate the association between workplace aggression and outcomes related to health and well-being.

3b. Strong psychosocial safety climates and ethical infrastructures will reduce the negative impact of workplace aggression on the health and well-being of employees.

Specific hypotheses about the relationships between variables, causal associations, and moderator and mediating variables will be developed and tested in individual studies based on data from the current project.

## Materials and Methods

### Reference Group

A reference group was set up to advise on the project. The reference group comprised stakeholders with knowledge about child protection work. The members were recruited from Oslo municipality (including first-line employees, managers, and safety representatives) and from the major union for child protection workers (The Norwegian Union of Social Educators and Social Workers). The main tasks of the reference group in the implementation phase of the project were to provide suggestions for the study questionnaire and to oversee the practical implications of the project.

### Study Design

This project applies two different study designs. Firstly, all employees in the child welfare service in Oslo municipality will be invited to participate in a prospective questionnaire survey that includes three measurement points over 12 months. The questionnaire will be identical at all three assessments. This kind of longitudinal design allows for (1) establishing the sequence of events; (2) following change over time in particular individuals within the cohort; (3) excluding recall bias in participants by collecting data prospectively and prior to knowledge of a possible subsequent event occurring; and (4) the ability to correct for potential cohort effects (Caruana et al., 2015). Hence, compared to cross-sectional designs, the utilized study design allows for determining directions of the studied associations and thereby provides indications about causal relationships. A time lag of 6 months between the survey time points is applied in order to reduce the risk of recall bias, which is likely to be present in longer intervals. Data will be collected at the work unit and team levels, enabling a multilevel approach to data analysis.

**FIGURE 2:**
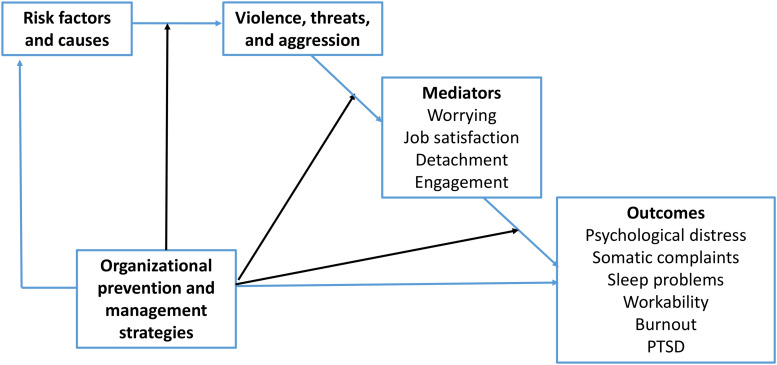
Time plan and activities for the research project.

Secondly, respondents who give their consent in the questionnaire survey will be contacted and asked to also participate in a quantitative diary study. The diary study will be conducted electronically using a web-based questionnaire that can be filled out using a smartphone, tablet, or a computer. The purpose of the diary study was to map day-to-day variations in employee emotions and how these emotions are influenced by exposure to threats and violence related to work. A graphical overview of the study design is presented in Figure 2.

### Ethical Approval and Consent to Participate

The project will be conducted in accordance with the World Medical Association Declaration of Helsinki. The Regional Committees for Medical and Health Research Ethics (REC) in Norway (REC South East) have approved this project (project no. 28496). In line with the General Data Protection Regulation (GDPR), the National Institute of Occupational Health (NIOH) has acquired permission from the Norwegian Centre for Research Data (NSD; approval: 226309) to process the personal data in this project for research purposes. Following the approval from REC, the project will be finished by 31 December 2024. Anonymized data will be available for use until 31 December 2029. All participants who are included in the questionnaire survey and the diary study will sign an informed consent before participation. This procedure for securing informed consent was approved by the ethics committee.

All data will be collected through the *Resource center for psychological and social factors at work*, developed by the NIOH in Norway. This is a web-based system for the secure administration of questionnaires. The system is developed for the purpose of tracking individuals over time and to couple data to registries in a way that satisfies the demands for anonymity and personal security. When accessing the web-based questionnaire using a personal login code, the respondents need to confirm their informed consent before responding to the questionnaire. No personally identifiable information about the respondents will be available to researchers as data will be de-identified prior to analyses.

### Sample

In the prospective survey part of the project, all 1,500 employees in the child welfare services and childcare institutions in the Oslo municipality will be invited to participate. The survey will be conducted electronically (although paper forms will be available if needed). Based on previous studies from similar settings (Nielsen et al., 2009; Finne et al., 2011; Aagestad et al., 2014), the expected response rate is between 50 and 70% at each of the measurement points. The magnitude of the questionnaire will be reduced at survey points 2 and 3 in order to retain the response rate across all measurement points. Items will excluded on the basis of the responses on the first survey (e.g., through factor analysis). For the baseline measurement, the expected response rate will give a sample size in the area of 750–1,050 respondents. Across all three time points, the sample size will range from 190 to 515 respondents, depending on the response rate. Given a prevalence rate of 40% for workplace aggression, as is the case in earlier studies (Aagestad et al., 2015), the necessary sample size for a power of 0.95 and α = 0.05 in the analyses of two groups (exposed/non-exposed) with a dichotomous outcome variable is estimated to be 318^1^. Hence, by inviting 1,500 potential respondents with a response rate of at least 50%, it is likely that the needed sample size is achieved. Respondents with more than 10% missing data on the study variables will be excluded from the analyses. The remaining missing data will be handled with multiple imputation or with full information maximum likelihood (FIML) estimation with robust standard errors (Graham, 2009).

To be able to assess the impact of threats and violence, only employees that have face-to-face sessions with children, youths, or their relatives will be recruited for the diary study. In the first round of the questionnaire survey, employees will be asked whether they will give their consent to also participate in the diary study. The selection mechanism will thereby be self-selection as only those employees who are willing to participate are invited. The intake for the study will be closed when 150 employees are recruited. With 150 employees and 14 daily measurements, the diary study will collect a total of 2,100 measurement points. The information from the diary study will be linked to the survey data.

### Measures

The questionnaire contains items and inventories that can be classified into the following five main categories: (1) demographics and background information; (2) physical and psychological aggression; (3) psychosocial safety climate and ethical infrastructure; (4) psychosocial work factors; and (5) health and well-being. A complete overview of the included scales and inventories for the questionnaire survey is found in Table 1, whereas the inventories for the diary study are presented in Table 2. In the following, we will present the assessment of the demographic factors, violence, threats, and aggression and the indicators of the prevention and management of violence and aggression. The scales included to assess the psychosocial work factors and health and well-being are all well-established instruments with previously demonstrated psychometric properties and therefore will not be further described in this protocol (please see the cited studies for information about these inventories).

**TABLE 1:** Overview of inventories in the main survey questionnaire.

Topic	Variables	Method	No. of items	Reference(s)
Background	Demographics	Single items	16	
Aggression	Violence and threats	Single item	9	
	Violence, threats, and aggression	Scale	21	Barling et al., 2001; Gadegaard et al., 2018
	Physical injuries following violence	Scale	11	Developed for survey
	Safety and personal competence	Single items	6	Nielsen et al., 2015a
	Online harassment	Scale	8	Developed for survey
	Exposure to bullying behavior	Scale	9	Notelaers et al., 2018
	Power imbalance bullying	Scale	5	Nielsen et al., 2017b
	Workplace bullying	Single item	1	Einarsen and Skogstad, 1996
Psychosocial safety climate and ethical infrastructure	Conflict management climate	Scale	9	Rivlin, 2001
	Violence management climate	Scale	4	Rivlin, 2001
	Psychosocial safety climate	Scale	3	Hall et al., 2010
	Procedures for reporting threats and violence	Scale	4	Developed for survey
	Training with regard to handling threats and violence	Single items	7	Developed for survey
	Violence prevention behavior	Scale	9	Gadegaard et al., 2018
Psychosocial work factors	Job demands and control	Scale	10	Van Veldhoven and Meijman, 1994
	Role conflict and role clarity	Scale	7	Dallner et al., 2000; Wannstrom et al., 2009
	Intragroup conflict	Scale	8	Jehn, 1995
	Task and person conflicts	Scale	5	Skogstad et al., 2007
	Emotional dissonance	Scale	4	Zapf et al., 1999
	Constructive leadership	Scale	3	Ekvall and Arvonen, 1994
	Perceived supervisor support	Scale	3	Dallner et al., 2000
	Laissez-faire leadership	Scale	4	Bass and Avolio, 2004; Nielsen et al., 2019
	Trust in colleagues	Scale	6	Developed for survey
	Job–family balance	Scale	5	Matthews et al., 2010; Haslam et al., 2015
Health and well-being	Psychological detachment	Scale	4	Sonnentag and Fritz, 2007
	Job satisfaction	Scale	4	Brayfield and Rothe, 1951; Hetland et al., 2008
	Intent to leave	Scale	3	Sjoberg and Sverke, 2000
	Work engagement	Scale	3	Schaufeli et al., 2017
	Work-related worries	Scale	11	Adapted from Larsen et al., 2009
	Anxiety and depression	Scale	17	Derogatis et al., 1974
	Client-related burnout	Scale	6	Kristensen et al., 2005
	Cognitive complaints	Scale	4	Kristensen et al., 2005
	Emotional exhaustion and depersonalization	Single items	2	West et al., 2009
	Somatic complaints	Single items	4	Steingrimsdottir et al., 2004
	Posttraumatic stress	Scale	6	Thoresen et al., 2010
	Insomnia	Scale	7	Pallesen et al., 2008
	Trait anger	Scale	5	Forbes et al., 2014
	Concerns about Covid-19 situation	Single item	1	Developed for survey

**TABLE 2:** Overview of inventories in the diary study.

Variables	Method	No. of items	Reference(s)
Violence and threats	Single item	2	
Workplace bullying	Single item	1	
Positive and negative affects	Scale	10	Watson and Clark, 1994
Sleep quality	Single item	1	
Work engagement	Scale	3	Schaufeli et al., 2017
Psychological detachment	Scale	4	Sonnentag and Fritz, 2007
Emotional exhaustion and depersonalization	Single items	2	West et al., 2009
Perceived supervisor support	Scale	3	Dallner et al., 2000

#### Background Information and Demographics

The background and demographic factors that will be recorded in the survey include age, gender, marital status, employment status (part- vs. full-time), seniority, education, shift work, leadership responsibility, position as a union representative, and number of child protection cases worked with during the last 6 months.

#### Physical and Psychological Aggression

Exposure to physical violence and threats of violence from children, youths, and their relatives will be assessed using a 21-item questionnaire. The majority of the items are taken from two established indicators for assessing workplace violence (Barling et al., 2001; Gadegaard et al., 2018), whereas some were developed for this study in order to fully capture the forms of violence that child protection employees can experience. The added items were based on suggestions from the reference group. This inventory captures exposure to specific forms of violence rather than to more general categories, such as those found in other scales like the Perception of Aggression Scale (Palmstierna and Barredal, 2006; De Benedictis et al., 2012). The utilized scale will be psychometrically tested and validated as a part of the current project. The respondents are asked to indicate their exposure during the last 6 months before the survey. Example items are: “Been cornered or placed in a position that was difficult to get out of” and “Someone threatened to kill you.” The response alternatives are: “never,” “once,” “twice,” “three times,” “four times,” and “five or more times.” In addition, the respondents will be asked several single-item questions related to threats and violence, including questions about the perpetrator, previous exposure to threats and violence, threats to family members, perceptions of physical safety, and perceived competence to deal with threats and violence.

Exposure to online harassment and threats will be surveyed using eight items developed for this study. These items were developed through discussions with employees in the child protection service and with the reference group for this project. After explaining that the context of the questions is online media, the respondents are asked to indicate their agreement with the included items using a 6-month time frame. Example items are: “Received messages with threats” and “Having personal information shared without your consent.” Responses will be given on a five-point scale ranging from “never” through “rarely,” “sometimes,” “often” to “very often.”

Exposure to psychological aggression from colleagues in the workplace is measured with the nine-item version of the *Negative Acts Questionnaire—Revised* (NAQ-R) inventory (Einarsen et al., 2009). NAQ-R describes negative and unwanted behaviors that may be perceived as bullying if occurring on a regular basis. This inventory assesses multiple forms of psychological aggression, including verbal abuse, social exclusion, slander, and humiliation. All items are formulated in behavioral terms and focus on the mere exposure to inappropriate behaviors while at work, with no references to the term “bullying” (Einarsen and Nielsen, 2015). Example items are “Spreading of gossip and rumors about you” and “Being shouted at or being the target of spontaneous anger or rage.” The respondents are asked to indicate how often they have been exposed to each specific item in the questionnaire at their present worksite during the last 6 months. The response categories range from 1 to 5 (“never,” “now and then,” “monthly,” “weekly,” and “daily”).

Building on the study findings by Nielsen et al. (2017b), power imbalance with regard to the bully will be measured with a five-item scale developed for this project. Referring to the items in the NAQ, the scale asks about whether respondents exposed to bullying behavior experience an imbalance of power opposite the perceived bully. An example item is “Felt a sense of hopelessness and resignation in relation to what you have experienced.” Responses are given on a scale ranging from “never,” through “sometimes,” “occasionally,” “often,” to “every time.”

Self-labeled victimization from workplace bullying will be measured with a single item used in several previous studies on bullying (Olweus, 1991; Einarsen and Skogstad, 1996; Solberg and Olweus, 2003; Nielsen et al., 2011). After being presented with the following definition – “*Bullying (harassment, badgering, niggling, freezing out, offending someone) is a problem in some workplaces and for some workers. To label something bullying it has to occur repeatedly over a period of time, and the person confronted has to have difficulties defending himself/herself. It is not bullying if two parties of approximately equal ‘strength’ are in conflict or the incident is an isolated event*” (Einarsen and Skogstad, 1996, p. 191) – the respondents are asked, “Have you been subjected to bullying at the workplace during the last 6 months?” The response categories are “no,” “rarely,” “now and then,” “once a week,” and “several times a week.”

#### Psychosocial Safety Climate and Ethical Infrastructure

Nine items adapted from the Conflict Management Climate Scale (Rivlin, 2001) will measure climate for conflict management and climate for violence management. The scale assesses the fairness of the dispute resolution and was designed to measure the perceived quality of the organizational procedures and managers’ abilities to handle interpersonal conflicts, bullying, and harassment. For four items, the wording was retained from the original scale in order to reflect the management procedures for conflict, whereas the wording for five items was changed in order to reflect the management procedures in cases of threats and violence. Example items are: “We have good procedures and methods for resolving disagreements and conflicts at my workplace” (conflict management climate) and “The management handles cases of violence and threats well” (violence management climate). Three items from the Psychosocial Safety Climate Scale (Hall et al., 2010) are included to assess the respondents’ perceptions regarding the organizations’ policies, practices, and procedures for the protection of workers’ psychological health and safety. An example item is “Employees’ psychological health is taken seriously at my workplace.” *Violent prevention behavior* at the top management, supervisor, and co-worker levels will be measured with the nine-item scale developed by Gadegaard et al. (2018). Example items are “Invests a lot of time and money in violence-prevention training for workers” (top management), “Your supervisor encourages staff to report physical violence” (supervisor), and “Your co-workers give sufficient help and support after a violent or threatening incident” (co-worker). A five-point Likert scale from 1 (“do not agree”) to 5 (“agree completely”) will be used for the responses for all items related to the above scales.

The effectiveness of the procedures for reporting exposure to threats, violence, bullying, and other inappropriate behavior at the workplace will be assessed with the four-item scale developed for this project. An example item is “We have good reporting procedures that should be used when one has been subjected to violence.” The response categories for this scale are given on a five-point Likert scale from 1 (“do not agree”) to 5 (“agree completely”). “Do not know” was added as a response alternative for respondents not familiar with reporting procedures.

Training with regard to handling threats, violence, and aggression will be surveyed using five single-item questions asking about whether or not the respondents have received training during the last 2 years with regard to (a) threats and violence, (b) conflicts in general, (c) bullying and harassment, and (d) sexual harassment. The response alternatives are “No,” “Yes,” and “Yes, but more than 2 years ago.” In addition, the respondents are asked about whether they are satisfied with the quality of the training.

### Registry Data

Providing informed consent from participants, the survey data will be linked to registry data on sickness absence from the employer. The data on sickness absence will include the total number of episodes with absence and the total number of days with absence during the last year before the survey and throughout the survey period.

### Data Analysis and Statistics

Following the described aims, this project will determine the prevalence rates; group differences; and direct, indirect, and conditional associations between the study variables both cross-sectionally and over time. Group differences will be tested with chi-square tests and ANOVA. Associations between variables will be examined using correlation- and regression-based approaches. Indirect and conditional effects will be analyzed with PROCESS, which is an observed variable ordinary least squares (OLS) and logistic regression path analysis modeling tool (Hayes, 2013), as well as with structural equation models. Longitudinal associations between variables will be adjusted for stability in variables in order to model changes over time (Little, 2013). In order to capture the multilevel structure of the quantitative diary study data, where the daily measurements (level 1) of the study constructs are nested within individuals (level 2), multilevel analyses will be carried out using MLwiN and Mplus software packages. Associations with registry data will be analyzed with a modified model for count data, the negative binomial hurdle (NBH) model. This analysis is capable of capturing both overdispersion and excess of zero values (Mullahy, 1986). The NBH model analyzes data in two steps: (1) a log-binomial regression analysis which estimates the risk ratio of having at least 1 day of medically certified sickness absence vs. none and (2) a zero-truncated negative binomial analysis which produces incidence rate ratios for the number of days absent among the subsample being at least 1 day absent (Indregard et al., 2017). Potential control variables and confounders for the adjusted models, e.g., demographic variables, will be considered only when theoretically applicable (Spector and Brannick, 2011).

## Discussion

This project will address some important knowledge gaps in research on workplace aggression. Firstly, by using behavioral inventories to assess specific forms of physical and psychological aggression, the findings will provide prevalence estimates as well as an in-depth understanding of how these forms of aggression influence individuals and organizations. Secondly, whereas previous studies on workplace aggression have mainly used cross-sectional data (Neall and Tuckey, 2014; Rudkjoebing et al., 2020), the present project is based on longitudinal research designs, including a quantitative diary study, which is a novel approach within this field of research. Thirdly, by examining mediating and moderating variables in the relationship between aggression and outcomes, this project will generate novel knowledge important for extending and developing the theoretical basis of our understanding of workplace aggression, which is critical in order to adequately design upcoming studies. Finally, the current project will elucidate whether strong psychosocial climates and ethical infrastructures are beneficial with regard to protecting employees against the detrimental effects of workplace aggression. The resulting knowledge will aid efforts to improve public health and the overall quality of life of child welfare workers, as well as for other employees working with customers and clients. By identifying modifiable moderators at the organizational level that protect against the adverse consequences of aggression, the findings can be used to develop appropriate prevention and intervention programs. Being able to alleviate the impact of workplace aggression on health and well-being can significantly reduce the costs related to sickness absence, productivity loss, turnover, and disability retirement. The project will thereby have major benefits for individuals, organizations, and society at large.

### Strengths and Limitations

The project has several strengths. Through a prospective design, the data are based on repeated measurements of workplace aggression, intervening variables, and outcomes. This provides more reliable information about exposures (independent variables) than what has been common until now. As conducting an experimental study on overt workplace aggression is ethically problematic, prospective designs are the strongest form of scientific evidence for the causal association between aggression and potential effects. Another strength is that the survey builds on well-established and standardized inventories, psychometrically tested for validity and reliability. The included registry data are objective data and the data structure allows for multilevel models, where individual-level data can be aggregated to department and organizational levels. The selection of respondents is based on a probability mechanism as all child welfare workers in the surveyed municipality are invited to participate. Depending on the response rate, it is likely that the sample will be representative of the population.

There are also some limitations of the planned project. The included survey instruments are all self-report measures and the project is thus subject to limitations specific to self-report instruments, such as response-set tendencies. The survey data are measured from the same source. As such, common method variance may inflate the relations between constructs (Podsakoff et al., 2003). However, the use of a longitudinal design as well as the opportunity to obtain co-worker reports of working conditions at the team and work unit levels should limit the risk of common method variance caused by self-report biases (Caruana et al., 2015). The main survey questionnaire is quite extensive and the time needed to participate may inflate the attrition and dropout rates.

### Assessment of Potential Bias

#### Response Bias

Ill health can be associated with non-response (Drivsholm et al., 2006), and it can be questioned whether the final sample is representative of the overall population or survey pool. However, research indicates that this health bias is not a major problem in occupational health research (Nielsen and Knardahl, 2016). Due to their personal experiences, employees may be more inclined to participate in the survey and the quantitative diary study if they have been exposed to aggression or mistreatment at the workplace. This situation is likely to inflate the prevalence estimates found in our sample in case of substantial non-response among non-exposed workers. We attempt to evade this problem by informing the respondents of their value to the study even though they have not experienced aggression. To motivate all employees and managers to participate, they will be informed about how the survey findings can be used as a tool to assess and improve working conditions. The strict procedures for the confidential treatment of data will be emphasized. Moreover, non-respondents will receive several reminders during the survey collection period.

#### Recall Bias

As is common with questionnaire surveys asking about a person’s past experiences, there is a risk of recall bias. To minimize this risk, we have included a relatively short and specific time period for most items (the last 6 months before the survey), and the items have a relatively low level of abstraction and should therefore be likely to be associated with specific events. As aggression represents a violation of a person’s physical and psychological integrity, people are likely to remember such events.

#### Selection Bias

As all employees in the child welfare services and childcare institutions in the Oslo municipality are invited to participate in the survey, the risk of selection bias is low.

### Dissemination

The prospective associations between workplace aggression and employee functioning, including findings on the prevention and management strategies, will be submitted to international peer-reviewed scientific journals. The results will be presented at national and international conferences. Oslo municipality will be informed about the findings through a series of feedback meetings. Central decision makers will also be informed about the findings.

## Conclusion

Given the limited evidence for effective strategies in preventing and managing workplace aggression, this study is important and timely and may help identify specific risks that child welfare workers are exposed to, the relative impact of this kind of exposure on the health and well-being when compared with other risk factors, and the best ways of managing the problem. The project should thereby inform the sector with regard to actions in the form of intervention programs.

## Ethics Statement

The project will be conducted in accordance with the World Medical Association Declaration of Helsinki. The Regional Committees for Medical and Health Research Ethics (REC) in Norway (REC South East) have approved this project (project no. 28496). In line with the General Data Protection Regulation (GDPR), the National Institute of Occupational Health (NIOH) has acquired permission from the Norwegian Centre for Research Data (NSD; approval: 226309) to process the personal data in this project for research purposes.

## Author Contributions

MN was responsible for the writing of the manuscript. All authors contributed to the idea development, project design, and article, and approved the submitted version.

## Conflict of Interest

The authors declare that the research was conducted in the absence of any commercial or financial relationships that could be construed as a potential conflict of interest.

## Footnotes

1 https://www.dssresearch.com/resources/calculators/sample-size-calculator-percentage/
